# *Pengzhongiella daicongchaoi* gen. et sp. n., a remarkable myrmecophile (Staphylinidae, Pselaphinae, Batrisitae) from the Gaoligong Mountains

**DOI:** 10.3897/zookeys.326.5933

**Published:** 2013-08-22

**Authors:** Zi-Wei Yin, Li-Zhen Li

**Affiliations:** 1 Department of Biology, College of Life and Environmental Sciences, Shanghai Normal University, 100 Guilin Road, Shanghai, 200234, P. R. China.

**Keywords:** Coleoptera, Batrisini, taxonomy, new genus, new species, myrmecophily, *Pengzhongiella daicongchaoi*, *Odontomachus monticola*, Yunnan, China

## Abstract

A new genus and species, *Pengzhongiella daicongchaoi*, of the subtribe Batrisina (Batrisitae: Batrisini) is described from the Gaoligong Mountains, Yunnan, Southwest China. Adults were collected in a colony of *Odontomachus monticola*, and presented reduction of certain external characters and elongate appendages relating to myrmecophily. Description and illustrations of the habitus and major diagnostic features of the new taxon are provided; a brief discussion of its taxonomic placement is included. The new species also represents the first record of a Pselaphinae in association with an *Odontomachus* ant.

## Introduction

Currently in China, eleven genera of the subtribe Batrisina (Batrisitae: Batrisini) were reported to contain myrmecophilous species. These species were found inhabiting colonies of the ant genera *Lasius* Fabricius (*Batrisus* Aubé, *Dendrolasiophilus* Nomura, *Sinotrisus* Yin & Li, *Songius* Yin & Li, *Tangius* Yin & Li [uncertain record]), *Formica* Linnaeus (*Batrisodes* Reitter, *Sinotrisus*), *Myrmica* Latreille (*Hingtoniella* Jeannel, *Myrmicophila* Yin & Li), *Vollenhovia* Mayr (*Cerochusa* Yin & Nomura), and *Pachycondyla* Smith (*Tribasodes* Jeannel, *Tribasodites* Jeannel) (Besuchet 1979; Löbl and Besuchet 2004; Yin et al. 2010, 2011a, 2011b, 2012a, 2012b, 2012c; Yin and Li 2013a, 2013b; Zhao et al. 2010a, 2010b).

The general appearance of some Chinese myrmecophilous batrisines (e.g. *Batrisus*, *Dendrolasiophilous*, *Songius*, *Tangius*) tends to be more stout, with the antennae often compressed, smooth body surface, and reduction/loss of foveae, sulci, and carinae. While some species may not show particular morphological adaptions to myrmecophily (e.g. *Batrisodes*, *Sinotrisus*).

During a recent (April 2013) expedition made to the south Gaoligong Mountains in Yunnan, South China, an unusual batrisine was collected from a nest of *Odontomachus monticola* Emery, and presented remarkably elongate appendages and reduced foveae on the head, pronotum, and elytra. Despite the inadequate knowledge of the Asian Batrisitae, this species is readily recognized as new, though it cannot be placed in any described genus. We here establish a new genus, *Pengzhongiella* gen. n., for this unusual beetle, and provide a formal description. So far, no pselaphine has been recorded to live with *Odontomachus* ants.

## Material and methods

The type series is housed in the Insect Collection of  Shanghai Normal University, Shanghai, ChinaSNUC.

The collection data of the referred material are quoted verbatim. A slash (/) is used to separate different labels.

The terminology follows Chandler (2001), except for using ‘ventrite’ instead of ‘sternite’ when describing meso- and metathoracic structures.

Measurements are in millimeters. The following abbreviations are applied: AL length of the abdomen along the midline; AW maximum width of the abdomen; EL length of the elytra along the sutural line; EW maximum width of the elytra; HL length of the head from the anterior clypeal margin to the occipital constriction; HW width of the head across eyes; PL length of the pronotum along the midline; PW maximum width of the pronotum. Length of the body equals HL + PL + EL + AL.

## Taxonomy

### Pengzhongiella

Yin & Li gen. n.

http://zoobank.org/8F5265D2-9927-421F-ABEC-B246C3BD22F7

http://species-id.net/wiki/Pengzhongiella

Figs 1 – 3

#### Type species.

*Pengzhongiella daicongchaoi* sp. n. (here designated).

#### Diagnosis.

Head rectangular; lacking frontal rostrum. Pronotum with distinct lateral longitudinal sulci, disc barely convex; lateral antebasal foveae present; lacking median antebasal fovea, antebasal sulcus, antebasal tubercles, and basolateral foveae. Each elytron with three reduced basal foveae, lacking discal stria. Abdomen with basolateral foveae on tergites IV–VII; tergite IV the longest.

#### Description.

Length 2.02–2.18 mm. Head (Fig. 2A) rectangular; lacking frontal rostrum and frontal fovea, antennal tubercles indistinct; punctiform vertexal foveae nude, shallow U-shaped impression connecting foveae; with 11 antennomeres, clubs formed by apical three antennomeres (Figs 3A, B); lacking ocular-mandibular carinae; eyes rounded, with posteroventral margins shallowly emarginate; maxillary palpi with palpomeres II basally pedunculate, III nearly triangular, IV fusiform; gular foveae (Fig. 2B) in shared opening, linear gular carina slightly indicated.

**Figure 1.**
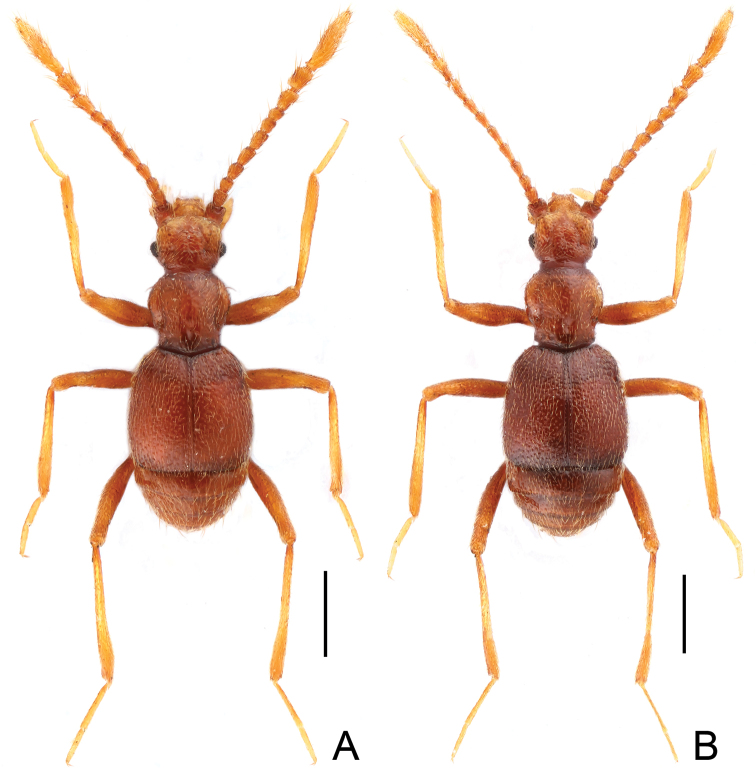
Habitus of *Pengzhongiella daicongchaoi*. **A** male **B** female. Scales: 0.5 mm

**Figure 2.**
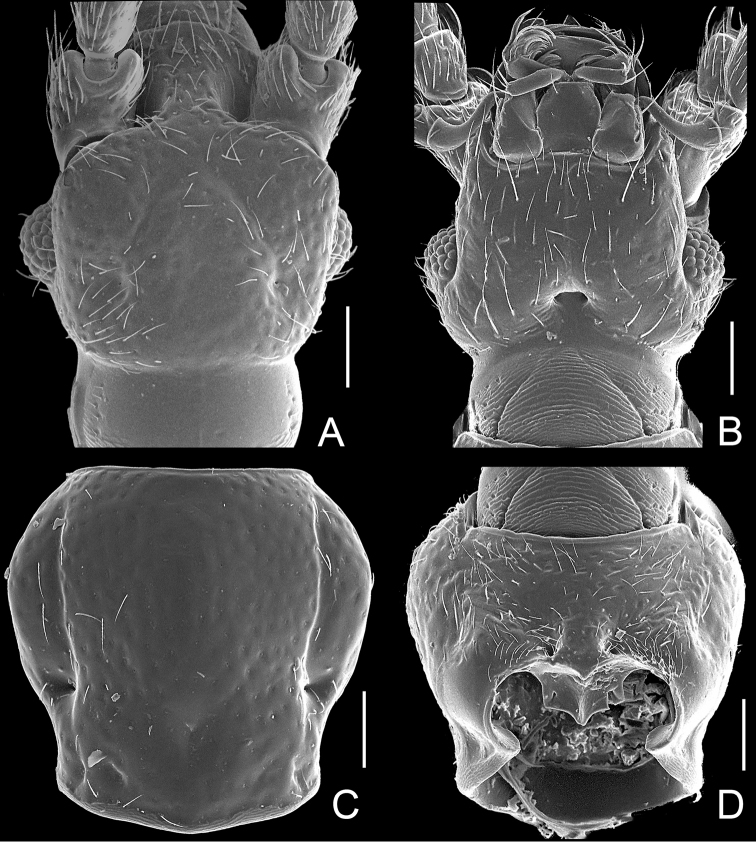
Diagnostic features of *Pengzhongiella daicongchaoi*. **A** head, in dorsal view **B** same, in ventral view **C** pronotum **D** prosternite. Scales: 0.1 mm.

Pronotum (Fig. 2C) with distinct lateral longitudinal sulci, lacking median longitudinal and antebasal sulci; small lateral antebasal foveae nude, lacking median antebasal fovea and antebasal spines; basolateral foveae absent, replaced by shallow impressions; lateral margins lacking spines. Prothorax (Fig. 2D) lacking paranotal sulci; with lateral procoxal foveae.

**Figure 3.**
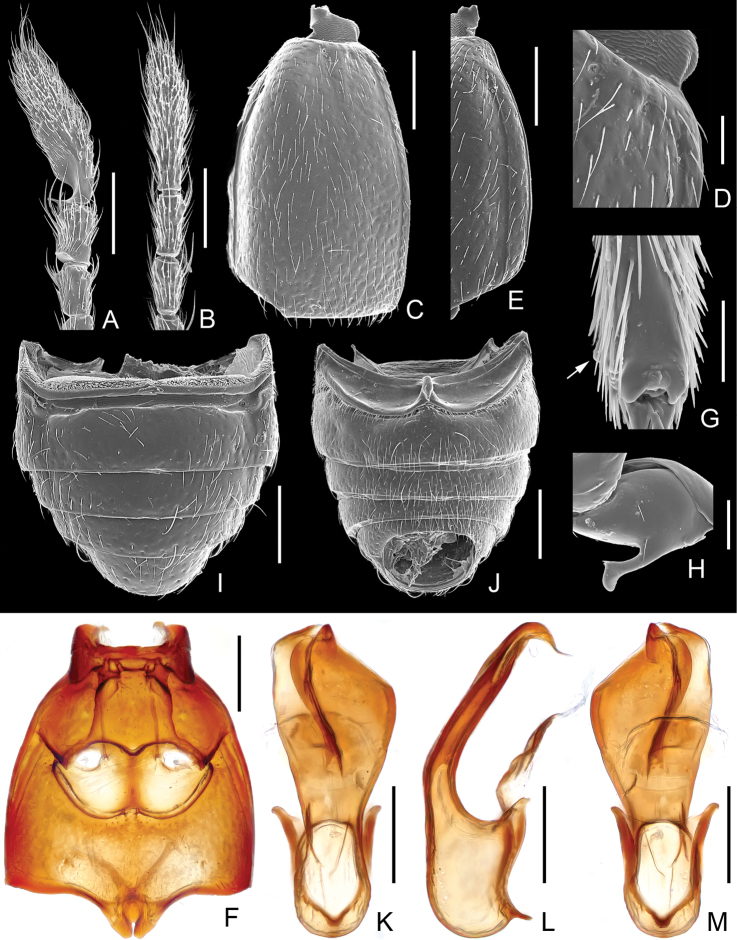
Diagnostic features of *Pengzhongiella daicongchaoi*. **A** male antennal club **B** female antennal club **C** left elytron **D** elytral base, enlarged **E** elytral lateral margin **F** Meso- and metaventrite **G** apical portion of mesotibia **H** metatrochanter **I** abdomen, in dorsal view **J** same, in ventral view **K** aedeagus, in dorsal view **L** same, in lateral view **M** same, in ventral. Scales (mm): **A, B, C, E, F, I, J** = 0.2; **K, L, M** = 0.1; **D, H, G** = 0.05

Each elytron with three punctiform basal foveae (Figs 3C, D), lacking discal stria; lacking subbasal foveae; sutural stria complete; subhumeral fovea present, with complete marginal stria (Fig. 3E); apicolateral margins broadly emarginate.

Mesoventrite (Fig. 3F) with lateral foveae forked for short distance, anterior fork as wide as median fork, median fovea widely separated, opening into shared transverse cavity; lateral mesocoxal foveae small; small lateral metaventral foveae close; metacoxae moderately separated; metaventrite with median sulcus extending to near apex, apex with narrow slit.

Tergite IV (first visible tergite) longest (Fig. 3I), deeply impressed across base, basolateral foveae in lateral endings of sulcus; lacking marginal carinae; V–VII subequal in length, lacking mediobasal sulcus, with basolateral foveae. Sternite IV (second visible sternite) longest (Fig. 3J), with mediobasal foveae at inner margins of shallow basolateral sulci, two pairs of small basolateral foveae present; sternite IV about twice length of V at midline, V–VII successively shorter, with pair of basolateral foveae.

First two pairs of tarsi with second and third tarsomeres subequal in length, metatarsi with second tarsomeres longer than third ones.

Males with antennae, mesotibiae, and metatrochanters modified. Aedeagus with dorsal lobe largely fused to median lobe, paramere connected with median lobe by membrane.

#### Comparative notes.

At this time *Pengzhongiella* cannot be placed near any genus, and seems to form an isolated group within Batrisina. The long appendages are rarely observed in Asian myrmecophilous batrisines. Coupled with the foveal pattern of the head, pronotum, and elytra, and other external characters, *Pengzhongiella* can be quickly separated from all known genera, especially the myrmecophilous members of the Asian Batrisitae. The exceptionally elongate antennae and legs are shared with the Sumatran *Akarbatrus* Löbl and the Australian *Mossman* Chandler. Both *Akarbatrus* and *Mossman* lack elytral basal foveaeand the basal impression of the tergite IV. The former has a sexually modified pronotum in the male, while *Mossman* has the pronotum lacking lateral longitudinal sulci, with two antebasal tubercles, and an outer pair of basolateral foveae (Chandler 2001, Löbl 2009). *Pengzhongiella* has three punctiform foveae at the base of each elytron, the pronotum has a pair of lateral sulci and small antebasal foveae, while other sulci and foveae are completely reduced probably due to the myrmecophily, and tergite IV has a thin, deep basal sulcus.

#### Etymology.

The new genus and species is named in honor of Zhong Peng and Cong-Chao Dai, respectively, for their collection of the type series. Gender of the generic name is feminine.

### Pengzhongiella daicongchaoi

Yin & Li sp. n.

http://zoobank.org/C1D36E04-F0AC-4C06-98EC-900B35348211

http://species-id.net/wiki/Pengzhongiella_daicongchaoi

Figs 1 – 3

#### Type material

(6 ♂♂, 35 ♀♀). **Holotype: CHINA:** ♂, labeled ‘China: W. Yunnan, Baoshan, S. Gaoligong Mt., Baihualing, ca. 42 km NE Tengchong, 25°117'36"N, 98°47'51"E, broad-leaved forest, nest of *Odontomachus monticola*, 1550–1600 m, 20.iv.2013, Peng & Dai leg. / Holotype [red], *Pengzhongiella daicongchaoi* sp. n., det. Yin & Li, 2013, SNUC’. **Paratypes: CHINA:** 5 ♂♂, 35 ♀♀, same label data as holotype, and all bear a paratype label as ‘Paratype [yellow], *Pengzhongiella daicongchaoi* sp. n., det. Yin & Li, 2013, SNUC’.

#### Description.

Male (Fig. 1A). BL 2.02–2.07 mm. Body reddish brown, maxillary palpi, tibiae, and tarsi lighter in color. Head, pronotum and elytra covered with fine setae. Head (Fig. 2A) as long as wide, HL 0.40–0.42 mm, HW 0.42–0.44 mm; vertex flat, vertexal foveae at level of posterior margins of eyes, connected by weakly indicated U-shaped impression; eyes moderately developed, each composed of about 40 facets; antennae greatly elongate, antennomeres X–XI (Fig. 3A) modified, XI longest, markedly concave basally. Pronotum (Fig. 2C) as long as wide, PL 0.46–0.47 mm, PW 0.47–0.48 mm; shallowly punctate; with rounded lateral margins; narrowed at base. Elytra (Fig. 3C) slightly wider than long, EL 0.66–0.67 mm, EW 0.71–0.72 mm; with rounded lateral margins. Metathoracic wings fully developed. Protibiae thickened (Fig. 1A); mesotibiae (Fig. 3G) bear tiny preapical tubercle; metatrochanters (Fig. 3H) greatly projecting at ventral margins. Abdomen wider than long, AL 0.50–0.51 mm, AW 0.64–0.65 mm, rounded at posterior margin. Aedeagus (Figs 3K–M) asymmetric, length 0.32 mm; basal bulb with large foramen; median lobe flattened dorso-ventrally; paramere weakly sclerotized.

Female (Fig. 1B). Similar to male in general. Measurements: BL 2.13–2.18 mm, HL 0.43–0.44 mm, PL 0.50–0.51 mm, PW 0.50–0.51 mm, EL 0.69–0.71 mm, EW 0.76–0.79 mm, AL 0.51–0.52 mm, AW 0.69–0.74 mm. Each eye composed of about 32 facets. Antennae simple. Metathoracic wings fully developed. Legs with protibiae narrower than those in male; mesotibiae lacking tubercle; metatrochanters simple. Width of genital complex 0.22 mm, slightly sclerotized, transverse.

#### Comparative notes.

The characteristic antennae and aedeagus, combined with the generic characters (see ‘Comparative notes’ of the genus), provide a quick separation of the new species from all other members of the Batrisini.

#### Distribution.

Southwest China: Yunnan.

#### Host ant and biology.

**Figure 4.**
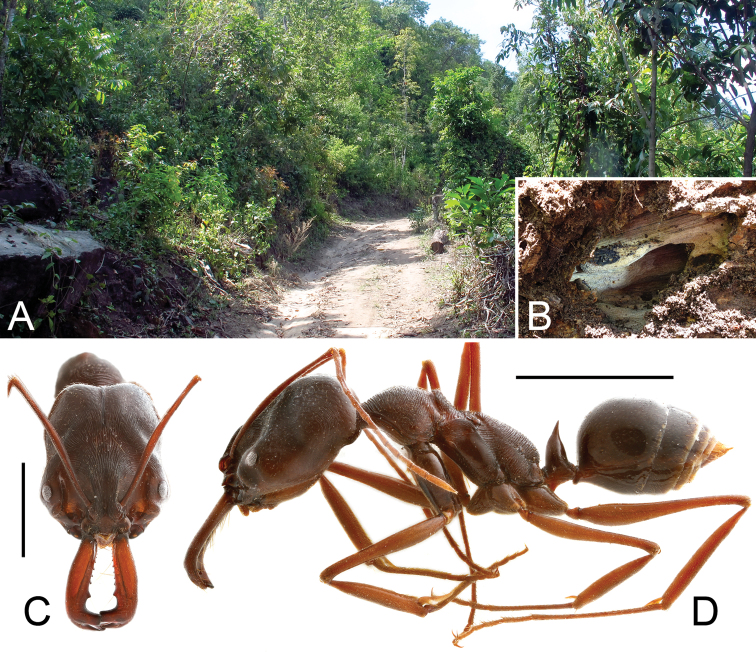
Habitat and ant host of *Pengzhongiella daicongchaoi*. **A** general habitat **B** nest of the host ant in a rotten tree **C** head of *Odontomachus monticola*, in frontal view **D** lateral habitus of *Odontomachus monticola*. Scales (mm): **C** = 3 mm; **D** = 5 mm.

All individuals of *Pengzhongiella daicongchaoi* were collected from a colony of *Odontomachus monticola* nested inside a rotten fallen tree, at the side of a road in an evergreen broad-leaved forest (Fig. 4A–D).It’s worthy of a note that a highly specialized species (lacking vertexal and pronotal median antebasal foveae) of *Batraxis* Reitter (22 ex.), one species of *Batrisoschema* Reitter (18 ex.), and one species of *Harmophorus* Motschulsky (5 ex.) were found in the same nest. Previously, no pselaphine has ever been reported living with members of the ant genus *Odontomachus*.

## Supplementary Material

XML Treatment for Pengzhongiella

XML Treatment for Pengzhongiella daicongchaoi
